# Clinical impact of high serum hepatocyte growth factor in advanced non-small cell lung cancer

**DOI:** 10.18632/oncotarget.17895

**Published:** 2017-05-16

**Authors:** Takahiro Tsuji, Yuichi Sakamori, Hiroaki Ozasa, Yoshitaka Yagi, Hitomi Ajimizu, Yuto Yasuda, Tomoko Funazo, Takashi Nomizo, Hironori Yoshida, Hiroki Nagai, Ken Maeno, Tetsuya Oguri, Toyohiro Hirai, Young Hak Kim

**Affiliations:** ^1^ Department of Respiratory Medicine, Kyoto University Graduate School of Medicine, Kyoto, Japan; ^2^ Department of Respiratory Medicine, Allergy and Clinical Immunology, Nagoya City University Graduate School of Medical Sciences, Nagoya, Japan

**Keywords:** hepatocyte growth factor, non-small cell lung cancer, c-MET, cytotoxic chemotherapy

## Abstract

Activation of c-MET through hepatocyte growth factor (HGF) increases tumorigenesis, induces resistance, and is associated with poor prognosis in various solid tumors. However, the clinical value of serum HGF (sHGF) in patients with advanced non-small cell lung cancer (NSCLC), especially those receiving cytotoxic chemotherapy, remains unknown. Here, we show that sHGF may be useful to predict tumor response and progression-free survival (PFS) in patients with advanced NSCLC. A total of 81 patients with NSCLC were investigated. sHGF levels were evaluated using ELISA at 4 time-points: at pre-treatment, at response-evaluation (1–2 months after treatment initiation), at the best tumor response, and at disease progression. As a control biomarker, CEA was also evaluated in lung adenocarcinoma. Positive-sHGF at response-evaluation predicted poor PFS compared with Negative-sHGF in both first-line (median, 153.5 vs. 288.0; *P* < 0.05) and second-line treatment (87.0 vs. 219.5; *P* = 0.01). In 55 patients that received cytotoxic chemotherapy, multiple Cox proportional hazards models showed significant independent associations between poor PFS and Positive-sHGF at response-evaluation (hazard ratio, 4.24; 95% CI, 2.05 to 9.46; *P* < 0.01). Lung adenocarcinoma subgroup analysis showed that in patients receiving second cytotoxic chemotherapy, there were no significant differences in PFS between patients with low-CEA compared with those with high-CEA, but Positive-sHGF at pre-treatment or at response-evaluation predicted poor PFS (35.0 vs. 132.0; *P* < 0.01, 50.0 vs. 215.0; *P* < 0.01, respectively). These findings give a rationale for future research investigating the merit of sHGF as a potential clinical biomarker to evaluate HGF/c-MET activity, which would be useful to indicate administration of c-MET inhibitors.

## INTRODUCTION

Hepatocyte growth factor (HGF) is a soluble ligand of the c-MET tyrosine kinase receptor. Activation of the HGF/c-MET signaling pathway contributes to the promotion of tumor cell motility, scattering, invasion, and metastasis, suggesting it to be a negative prognostic indicator for cell survival and recurrence in some solid tumors including lung cancer [1, 2].

Overexpression of both HGF and/or its receptor c-MET have been reported in non-small cell lung cancer (NSCLC) cell lines and patients [3–8]. Increased expression of HGF is associated with acquired resistance to EGFR-TKIs by bypass signaling via MAPK/ERK and PI3K/AKT pathways and by promoting clonal selection of subpopulations by c-MET amplification [9, 10]. Preclinical findings also showed that the aberrant c-MET/HGF pathway plays an important role in cytotoxic chemotherapy (CC) resistance in small-cell lung cancer (SCLC) [11].

Activation of the HGF/c-MET pathway has been evaluated using serum or plasma HGF, because HGF is expelled from tumor cells to the extracellular matrix and blood plasma by a paracrine mechanism [12]. Increased serum HGF (sHGF) has been reported to be a negative prognostic marker in various malignancies including colorectal cancer, gastric cancer [13–15], prostate cancer [16], ovarian cancer [17], breast cancer [18, 19], glioma[20], melanoma [21], and multiple myeloma [22, 23]. Recently, a Spanish group reported that high sHGF in patients with SCLC predicts poor outcome and epithelial-mesenchymal transition (EMT) change in the tumor [24, 25].

We previously reported that lung cancer cell lines had increased expression of c-MET due to gene amplification-induced cytotoxic drug resistance, and that resistant cells paracrine HGF and promote its resistance [26]. However, the clinical significance of sHGF in patients with advanced or recurrent NSCLC, especially in patients treated with CC, is yet to be identified. Here we report a retrospective cohort study suggesting that sHGF concentration is a potential predictive biomarker for poor clinical outcome in patients with NSCLC.

## RESULTS

### sHGF in NSCLC patients and healthy controls

**Figure 1:**
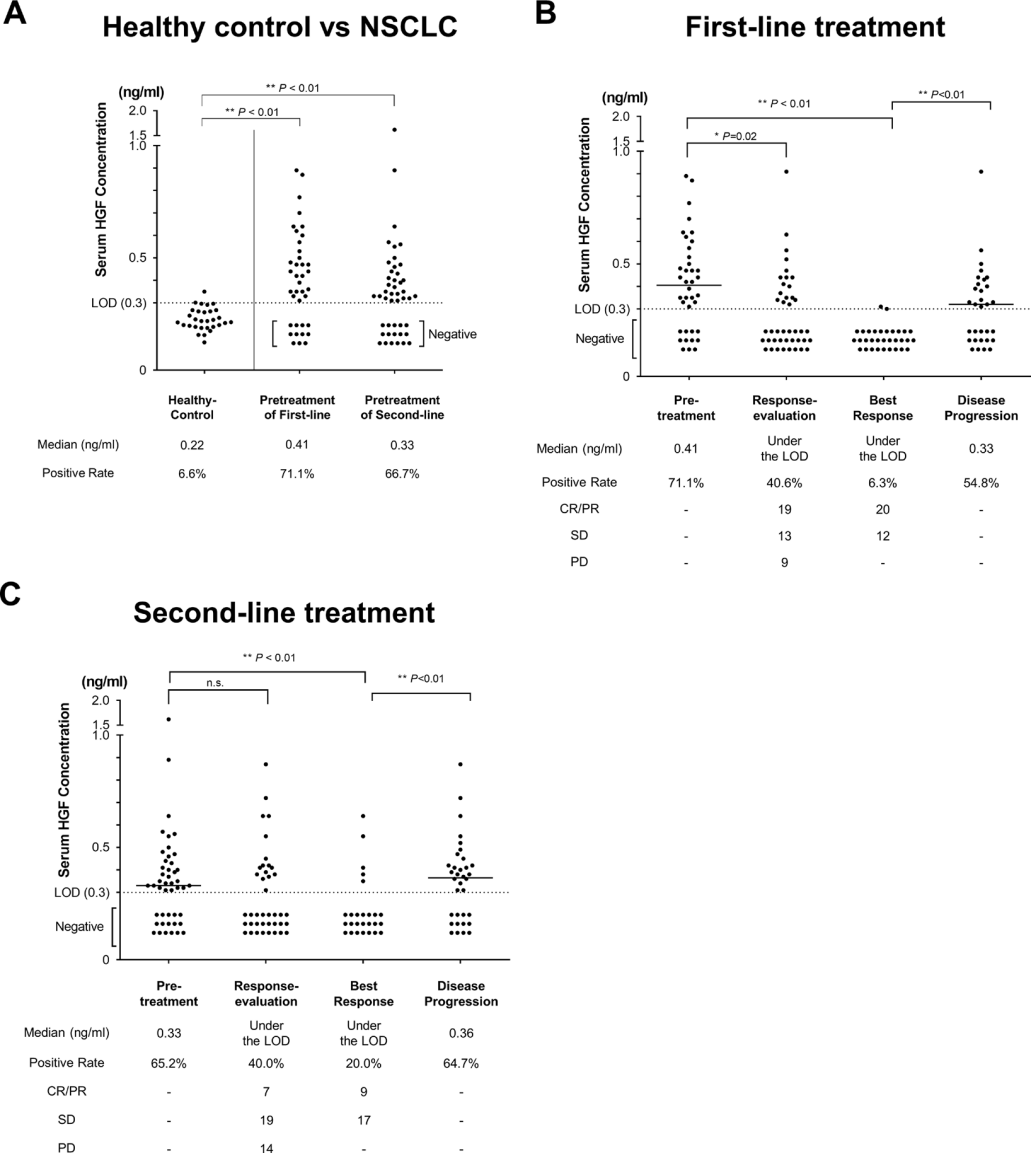
sHGF values in patients with NSCLC (**A**) sHGF values in healthy controls and patients with NSCLC. In healthy controls, values under the limit of detection (0.3 ng/ml) were extrapolated using a calibration curve. (**B**, **C**) The change in sHGF values in patients with NSCLC receiving first-line treatment (B) and second-line treatment (C). Black dots show the concentration of sHGF at 4 time points during treatment. sHGF medians, the rates of Positive-sHGF at each time point (positive rate), and the objective response at each time point are indicated below. In all Figures, the Mann-Whitney *U* test was used for comparisons.

A total of 81 patients were investigated; 53 patients received first-line and 48 patients received second-line therapy. The median sHGF value at pre-treatment of first-line and second-line therapy were 0.41 and 0.33 ng/ml, respectively (Figure 1A). Then, thirty healthy controls were collected and matched to the current study population by smoking status, gender, and age (Table 1). Of the 30, two healthy controls (6.7%) had Positive-sHGF. The value was significantly higher in patients with NSCLC compared with healthy controls (*P* < 0.01, using the Mann-Whitney *U* test, Figure 1A). In 28 healthy controls with Negative-sHGF, the values were extrapolated using a calibration curve to consider the rationale of the cutoff value (0.3 ng/ml). The median value was 0.22 ng/ml and the mean ± S.D. was 0.22 ± 0.05 ng/ml (Figure 1A).

**Table 1:** Characteristics of healthy volunteers

		Healthy volunteer control	NSCLC pts in first-line therapy	*P*-value	NSCLC pts in second-line therapy	*P*-value
*N*		30	53		48	
Sex	Female	16 (46.7%)	24 (45.3%)	0.48	21 (43.8%)	0.41
Age yr. (mean ± S.D.)		63.8 ± 7.0	65.6 ± 9.3	0.42	64.5 ± 11.8	0.85
Smoking	Never	11 (36.7%)	22 (41.5%)	0.66	21 (43.8%)	0.54
	Smoker	19 (73.3%)	31 (58.5%)		27 (56.3%)	

The characteristics of the healthy volunteers in this study. Data are presented as numbers. *P*-values were calculated using the chi-square test or Student's *t*-test in categorical variables or continuous variables, respectively. NSCLC: non-small cell lung cancer; pts: patients.

### sHGF trends in patients with NSCLC

In 53 patients receiving first-line therapy, Positive-sHGF was observed in 71.1%, 40.6%, 6.3%, and 54.8% at pre-treatment, response-evaluation, best response, and disease progression, respectively. sHGF concentration was significantly decreased at response-evaluation (median value (MV): under the limit of detection (LOD); *P* < 0.01) or at best response (MV: under the LOD; *P* < 0.01) compared with at pre-treatment (MV: 0.41 ng/ml). sHGF values at disease progression (MV: 0.33 ng/ml) were significantly increased compared with those at best response (*P* = 0.01) (Figure 1B). In 48 patients receiving second-line therapy, a similar trend was observed (Figure 1C). sHGF was decreased at best response compared with pre-treatment (MV: under the LOD vs. 0.33; *P* = 0.02) and increased at disease progression compared with best response (MV: 0.36 vs. under the LOD; *P* < 0.01). Then, sHGF kinetics in patients whose best responses were PD was investigated. sHGF values at response-evaluation (when PD) were not significantly increased compared with pre-treatment, but the values before next treatment tended to be increased (*P* = 0.06, Supplementary Figure 2A–2C).

### sHGF value was potentially associated with the best response

**Figure 2:**
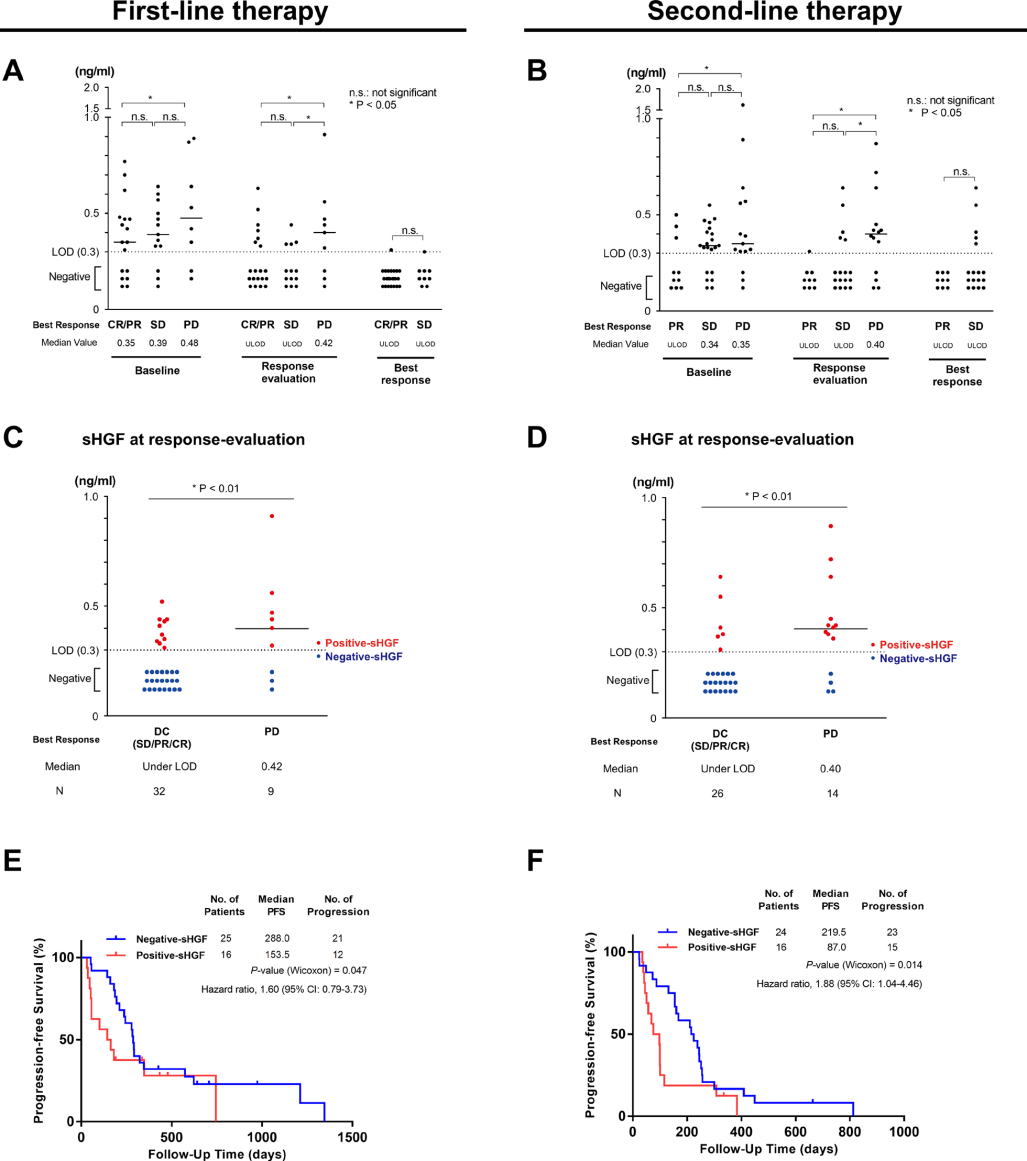
sHGF levels at response-evaluation predict progression-free survival (**A**, **B**) sHGF values according to the best response in patients that received first-line (A) and second-line (B) therapy. The *P*-value was calculated using the Mann-Whitney *U* test. (**C**, **D**) Serum hepatocyte growth factor (sHGF) levels at response-evaluation according to the achieved best response in first-line treatment (C) and second-line treatment (D). Red or blue dots indicate the sHGF value in each group and the bars show the median value. The *P*-value was calculated using the Mann-Whitney *U* test. (**E**, **F**) A Kaplan-Meier curve for progression-free survival according to sHGF levels at response-evaluation in patients with NSCLC receiving first-line treatment (E) and second-line treatment (F). The *P*-value was calculated using the Gehan-Breslow-Wilcoxon test. DC: disease control; PD: progressive disease; LOD: limit of detection.

Trends of sHGF value according to the best response were presented in Figure 2A and 2B. sHGF level at response-evaluation was significantly higher in patients whose best responses were PD compared with those whose diseases were controlled (CR, PR, or SD) (Figure 2C and 2D). sHGF values in patients with PR/CR tended to be lower compared with patients with SD, but a significant difference was not detected (Figure 2A and 2B).

### Positive-sHGF at response-evaluation predicted poor PFS in NSCLC

sHGF values at response-evaluation were obtained in 41 and 40 patients receiving first-line and second-line treatments, respectively (Table 2). PFS in Positive-sHGF patients was significantly shorter compared with Negative-sHGF patients in both first-line (Median PFS (days): 153.5 vs. 288.0; *P* = 0.047) and second-line (87.0 vs. 219.5; *P* = 0.01) treatments (Figure 2E and 2F). Patients with Positive-sHGF at diagnosis (pre-treatment of first-line) did not have significantly shorter PFS compared with patients with Negative-sHGF (*P* = 0.82) (Supplementary Figure 3C and 3D). In second-line treatment, the number of patients receiving EGFR-TKI was larger for Negative-sHGF compared with Positive-sHGF (Table 2). Subgroup analyses in both patients receiving EGFR-TKI and CC in second-line treatment showed that in both subgroups patients with Positive-HGF tended to have shorter PFS (Supplementary Figure 4).

**Table 2:** Patients’ characteristics in the current study

		First-line Treatment				Second-line Treatment			
		Total	Negative-sHGF at RE	Positive-sHGF at RE	*P*-value	Total	Negative-sHGF at RE	Positive-sHGF at RE	*P*-value
*N*		53	25	16		48	24	16	
Gender	Female	24 (45.3%)	14 (56.0%)	5 (31.3%)	0.20	21 (43.8%)	11 (45.8%)	8 (50.0%)	0.77
Age yr. (mean ± S.D.)		65.6 ± 9.3	64.8 ± 9.6	64.3 ± 9.6	0.88	64.5 ± 11.8	66.5 ± 11.2	59.1 ± 12.1	0.06
PS	≥ 2	6 (11.3%)	3 (12.0%)	1 (6.3%)	1.00	5 (10.4%)	2 (8.3%)	2 (12.5%)	1.00
	0–1	47 (88.7%)	22 (88.0%)	15 (93.8%)		43 (89.6%)	22 (91.7%)	14 (87.5%)	
Smoking	Never	22 (41.5%)	9 (36.0%)	6 (37.5%)	1.00	21 (43.8%)	15 (62.5%)	5 (31.3%)	0.11
	Smoker	31 (58.5%)	16 (64.0%)	10 (62.5%)		27 (56.3%)	9 (37.5%)	11 (68.8%)	
Histology	Ad	39 (73.6%)	20 (80%)	8 (50.0%)	0.11	41 (85.4%)	20 (83.3%)	13 (81.3%)	0.67
	Sq	10 (18.9%)	3 (12.0%)	6 (37.5%)		3 (6.3%)	2 (8.3%)	1 (6.3%)	
	Others	4 (7.6%)	2 (8.0%)	2 (12.5%)		4 (8.4%)	2 (8.3%)	2 (12.5%)	
EGFR mutation	Positive	23 (43.4%)	10 (40.0%)	5 (31.3%)	0.51	23 (47.9%)	15 (62.5%)	6 (37.5%)	0.19
	Wild type	26 (49.1%)	12 (48.0%)	10 (62.5%)		23 (47.9%)	8 (33.3%)	10 (62.5%)	
	Unknown	4 (7.6%)	3 (12.0%)	1 (6.3%)		2 (4.2%)	1 (4.2%)	0 (0%)	
Stage	IIIA	0 (0%)	0 (0%)	0 (0%)		3 (6.3%)	2 (8.3%)	1 (6.3%)	
	IIIB	4 (7.6%)	3 (12.0%)	0 (0%)		4 (8.3%)	1 (4.2%)	1 (6.3%)	
	IV	42 (79.3%)	17 (68.0%)	16 (100%)		35 (72.9%)	18 (75%)	12 (75.0%)	
	Recurrence	7 (13.2%)	5 (20%)	0 (0%)		6 (12.5%)	3 (12.5%)	2 (12.5%)	
Treatment	CC	36 (67.9%)	18 (72.0%)	13 (81.3%)	0.71	31 (64.6%)	11 (45.8%)	13 (81.3%)	*0.05
	EGFR-TKI	17 (32.1%)	7 (28.0%)	3 (18.8%)		17 (35.4%)	13 (54.2%)	3 (18.8%)	
Response	CR	1 (1.9%)	0 (0%)	0 (0%)		0 (0%)	0 (0%)	0 (0%)	
	PR	29 (54.7%)	13 (52.0%)	7 (43.8%)		11 (22.9%)	8 (33.3%)	1 (6.3%)	
	SD	14 (26.4%)	9 (36.0%)	3 (18.8%)		22 (45.8%)	12 (50.0%)	5 (31.3%)	
	PD	9 (17%)	3 (12.0%)	6 (37.5%)		15 (31.3%)	4 (16.7%)	10 (62.5%)	
ORR/DCR (best response)		56.6/83.0%	52.0/88.0%	43.8/62.5%		22.9/68.8%	33.3/83.3%	6.3/37.6%	

The characteristics of the patients with Positive-HGF and Negative-HGF at response-evaluation who received first-line or second-line therapy in this study. Data are presented as numbers. P-values were calculated using the Fisher's exact test or chi-square test, as appropriate, comparing Positive- and Negative-HGF patients. RE: response-evaluation; PS: performance status; Ad: adenocarcinoma; Sq: squamous cell carcinoma; LCC: large cell carcinoma; NSCLC: non-small cell carcinoma; EGFR: epidermal growth factor receptor; CC: cytotoxic chemotherapy; TKI: tyrosine kinase inhibitor; CR: complete response; PR: partial response; SD: stable disease; PD: progressive disease; ORR: objective response rate; DCR: disease control rate.

### sHGF was a predictive factor for PFS in patients that received cytotoxic chemotherapy

Next, clinical impact of sHGF in patients that received CC was investigated. In this retrospective cohort, fifty-five patients received CC. Cox proportional hazards models showed univariate associations (*P* < 0.2) between poor PFS and five factors; Positive-sHGF at response-evaluation, ECOG performance status (PS), EGFR-WT (or squamous cell carcinoma), second-line treatment, and monotherapy. A multivariate Cox proportional hazards model that included the five factors suggested that monotherapy and performance status were no longer significant factors (Supplementary Table 1). Monotherapy was highly correlated with second-line therapy (*P* < 0.01, Fisher's exact test, Supplementary Table 2). Then, the other four factors (sHGF, EGFR-WT, ECOG-PS, and second-line treatment) were included for the final multivariate Cox analysis, which detected a continued significant independent association between poor PFS and the sHGF value at response-evaluation (hazard ratio, 4.24; 95% CI, 2.00 to 9.25; *P* < 0.01) (Table 3). The backgrounds of the patients are presented in Supplementary Table 2.

**Table 3:** Univariate and multivariate analysis in patients that received cytotoxic chemotherapy using Cox proportional hazards model

N=55	Univariate analysis			Multivariate analysis	
Factor	HR (95% CI)	*P*-value		HR (95% CI)	*P*-value
Gender (Male / Female)	0.9 (0.51–1.63)	0.73			
Age yr (≥ 65 / < 65)	0.82 (0.47–1.46)	0.51			
Smoking (Smoker / Never Smoker)	1.2 (0.67–2.21)	0.55			
ECOG-PS (PS ≥ 2 / PS 0–1)	4.12 (0.94–12.84)	*0.06	>	1.45 (0.32–4.85)	0.59
Stage (IV / recurrence / III)	0.6 (0.28–1.59)	0.28			
Histology (Non-ad / Ad)	1.48 (0.8–2.64)	0.21			
EGFR-status (Negative or Sq / mutant)	2.3 (1.09–5.66)	*0.03	>	5.96 (2.29–17.85)	*< 0.01
Monotherapy (Monotherapy / Platinum Doublet)	2.06 (1.09–3.78)	*0.03			
Second-line (2nd-line / 1st-line)	2.01 (1.12–3.63)	*0.02	>	2.11 (1.14–3.94)	*0.02
sHGF at pre-treatment (Positive / Negative)	1.25 (0.68–2.26)	0.45			
sHGF at response-evaluation (Positive / Negative)	2.1 (1.18–3.73)	*0.01	>	4.24 (2–9.25)	*< 0.01

HR: hazard ratio; ECOG: Eastern Cooperative Oncology Group; PS: performance status; Ad: adenocarcinoma; EGFR: epidermal growth factor receptor; Sq: squamous cell carcinoma.

### sHGF was a potential predictive factor for PFS in patients after cytotoxic chemotherapy

**Figure 3:**
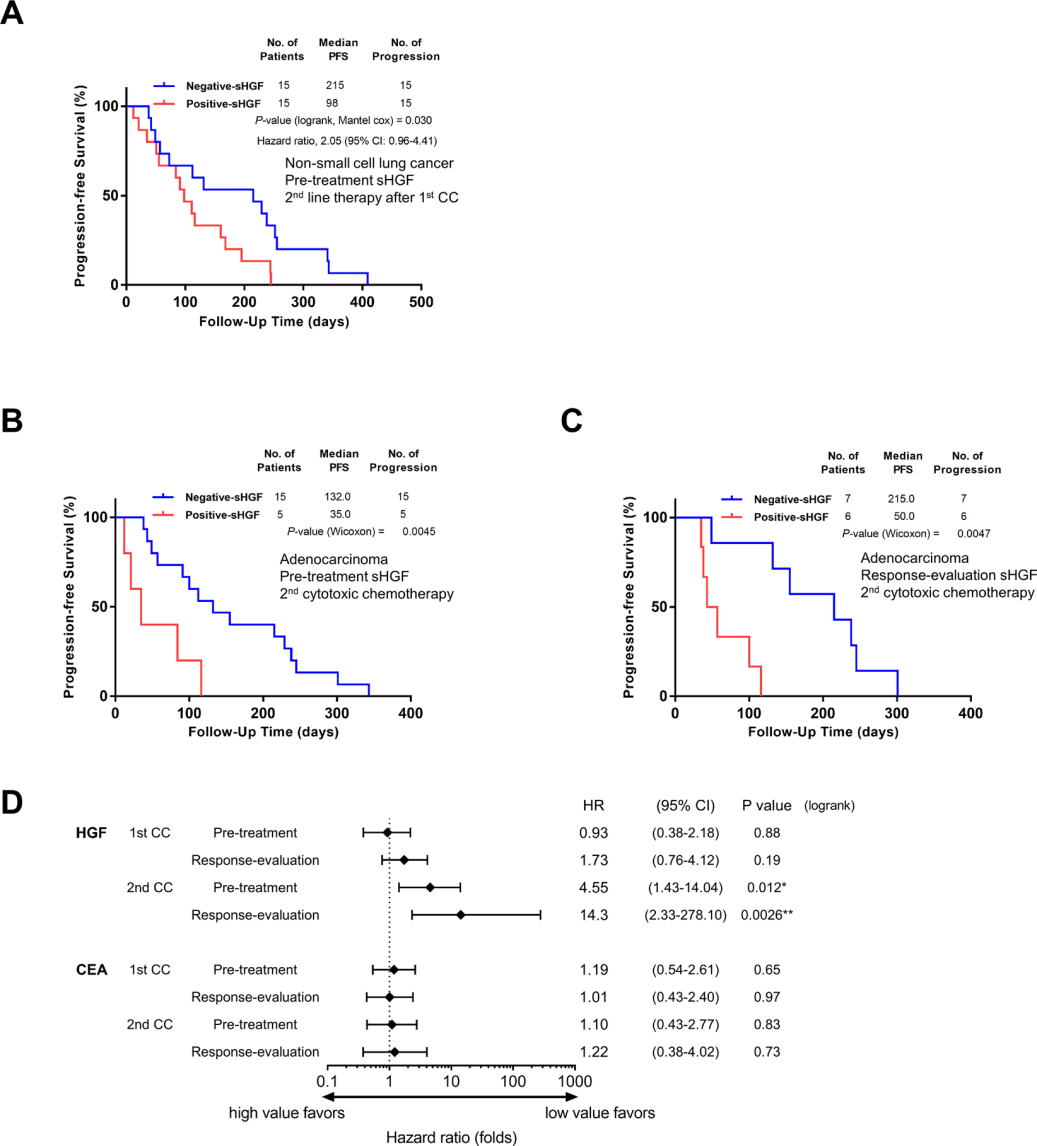
sHGF levels at a later time-phase predict progression-free survival in patients that received cytotoxic chemotherapy (**A**) A Kaplan-Meier curve for progression-free survival according to sHGF levels in patients that received second-line treatment after progression during first-line cytotoxic chemotherapy. The *P*-value was calculated using the Gehan-Breslow-Wilcoxon test. (**B**, **C**) A Kaplan-Meier curve for progression-free survival according to sHGF levels at pre-treatment (B) and at response-evaluation (C) in patients with lung adenocarcinoma receiving second cytotoxic chemotherapy. The *P*-value was calculated using the Gehan-Breslow-Wilcoxon test. (**D**) The hazard ratios (HR) for progression (black squares) and 95% CIs (horizontal lines). The HRs and *P*-values were calculated using the logrank test. The patients’ characteristics are presented in Supplementary Tables 4 and 5. HGF: hepatocyte growth factor; PFS: progression-free survival; 95% CI: 95 percent confidential interval; CC: cytotoxic chemotherapy; CEA: carcinoembryonic antigen.

Then, the clinical significance of sHGF in second-line treatment after the first-line CC was investigated. Thirty patients subsequently received second-line treatment after progression in first-line CC (Supplementary Table 3). Positive-sHGF after first-line cytotoxic chemotherapy (pretreatment of second-line therapy) predicted poorer PFS in second-line therapy compared with Negative-sHGF (median PFS, 98.0 vs. 215.0 days; HR, 2.05; *P* = 0.03 (logrank test), Figure 3A)

### sHGF was a predictive factor for PFS in patients with lung adenocarcinoma receiving cytotoxic chemotherapy

Finally, PFS in patients with lung adenocarcinoma treated with CC was examined by using sHGF values and a control marker, CEA. There were no significant differences in PFS or HR between patients with high-CEA compared with low-CEA at any time-points during the treatment course (Figure 3D, Supplementary Figure 5). In contrast, Positive-sHGF in patients receiving second CC predicted poorer PFS compared with Negative-sHGF both at pretreatment (median PFS, 35.0 vs. 132.0 days; HR, 4.55; *P* = 0.01, Figure 3B and 3D), and at response-evaluation (median PFS, 50.0 vs. 215.0 days; HR, 14.3; *P* < 0.01, Figure 3C and 3D). In first CC, Positive-sHGF at response-evaluation tended to predict shorter PFS, but a significant difference was not detected (median PFS, 163.0 vs. 281.5 days, *P* = 0.11). (Supplementary Figure 6)

## DISCUSSION

Here we reported that sHGF was associated with tumor response. Positive-sHGF at response-evaluation and at pre-treatment of the second CC also predicted poor PFS.

To date, the relationship between circulating HGF and clinical outcome has been reported in NSCLC. In early stage NSCLCs, sHGF was significantly higher compared with healthy controls [27] and predicted early recurrence after a standard operation [28, 29]. In patients treated with EGFR-TKIs, high circulating HGF predicted poor prognosis [30–33]. To the best of our knowledge, this is the first report to suggest the merit of sHGF as a potential biomarker in patients with advanced or recurrent NSCLC including populations receiving CC.

This trend of sHGF was concordant with the treatment effect (Figure 1B and 1C). Correspondingly, previous reports showed that the circulating HGF value was decreased after tumor resection in lung and breast cancer [18, 28], and was parallel with tumor response in SCLC [25]. sHGF protein is a tumor volume marker in patients with advanced or recurrent NSCLC. However, sHGF in patients with PD was not increased at response-evaluation and tended to be increased before the next treatment regimen, which suggested that the sHGF value was not just a volume marker.

Positive-sHGF at response-evaluation predicted early disease progression, but Positive-HGF at diagnosis did not. Similarly, previous reports showed that immunohistochemical overexpression of c-MET or high sHGF at diagnosis was not a prognostic factor in patients with NSCLC [31, 34]. As sHGF values correlate with tumor volume, the values at diagnosis would not predict early progression. In the current study, sHGF was potentially useful to predict PFS at a later time-point of the treatment course, although serum CEA was not (Figure 3C). It should be noted that Positive-sHGF can potentially identify treatment refractory patients at the start of the second CC. The sHGF value was not just a volume marker, but potentially predicted drug resistance.

Preclinical studies have explained the mechanism for increased sHGF in patients with poor PFS. HGF is a paracrine protein secreted by mesenchymal cells regulating cellular growth, motility, and morphogenesis, that acts as a multifunctional cytokine mainly in epithelial-origin cells [35]. Activation of HGF/MET autocrine in HGF- or MET-transgenic mice *in vivo* promoted hepatocarcinogenesis [36, 37]. We previously provided preclinical evidence that cytotoxic drug-resistant lung cancer cells secreted HGF and accelerated its resistance by increased expression of c-MET due to gene amplification, and that c-Met inhibitors restored cytotoxic drug sensitivity [26, 38]. A high sHGF value at later time-points of the treatment course would be associated with activity in the HGF/c-MET signaling pathway in the tumor.

For the clinical use of sHGF, it should serve as a marker for HGF/cMET activity. Companion diagnostics for MET inhibitors are needed, because a phase III trial failed to demonstrate that adding Onartuzumab, a MET inhibitor, to erlotinib improves overall survival in patients with NSCLC, although phase II and preclinical studies have suggest promising effects [39–42]. To date, immunohistochemistry of c-MET or genetic tests of MET have been used to evaluate HGF-c-MET activity [43]. However, there are several problems with these methods; repeated biopsy or frequent monitoring are complicated, small biopsy specimens from one lesion may not detect dominant resistant mechanisms in the whole tumor [44], and there are few chances to perform a re-biopsy in patients receiving CC. sHGF values are easy to monitor and suitable for determining dominant molecular alterations in the whole tumor. We hypothesize that the sHGF value could be used as a simple and repeatable activity marker for the HGF/c-MET pathway and provide a supplemental marker to indicate re-biopsy demonstrating HGF/c-MET activation.

A limitation of this study is its retrospective design and small sample size. Unexpected confounding biases and the influence of data deficiency cannot be excluded. Due to the supportive results in univariate and multivariate analyses, we believe that any bias would not influence our findings. Another limitation is that the correlation between sHGF values and c-MET activation was not investigated because re-biopsy for patients treated with CC is unusual. In future, activation of c-MET in patients treated with CC and showing a high sHGF value may merit further prospective investigation. Finally, at present, there is no conventional threshold for Positive-sHGF concentrations in patients with NSCLC. In previous studies, the threshold values for Positive-sHGF were approximately 1.9 ng/ml in SCLC [25], 2.4 ng/ml in gastric cancer [15], and 0.4 ng/ml in bladder cancer [45]. Mean sHGF values in healthy controls in previous reports also vary considerably (0.08 to 1.37 ng/ml) [25, 45–47]. In the current study, we used an ELISA kit which had been designed and approved for clinical use. The sHGF value in 200 Japanese healthy controls was reported as 0.19 ± 0.05 ng/ml (mean ± S.D.)[45]. According to historical controls of this kit and our healthy control study, the cutoff value was equivalent with an average +1.6 to +2.2 S.D. in healthy controls. The threshold might be different among tumor origins, histology, and the ELISA procedures. We believe that a standardized procedure and sharing of cutoff values is needed between clinics and translational laboratories.

In conclusion, the current study provided the first evidence that sHGF values were associated with the treatment response, and that the values at response-evaluation and at pre-treatment of the second CC are predictive markers of poor PFS in patients with NSCLC. These findings suggest future research investigating the merit of sHGF as a potential clinical biomarker to indicate administration of MET inhibitors in patients with NSCLC, or as a re-biopsy marker for patients treated with CC, may lead to the development of a new indicator of MET inhibitors and improve targeted therapies for lung cancer.

## MATERIALS AND METHODS

### Patients and clinical information

Between November 2013 and December 2015, serum samples from 81 patients with advanced cancer or receiving anticancer treatment were collected and cryopreserved at Kyoto University Hospital (Kyoto, Japan). Patients who were not evaluated objective response were excluded. Clinical information was obtained from electronic medical records at the institution. The study protocol had been prepared in accordance with the Declaration of Helsinki and was approved by the Kyoto University Graduate School and Faculty of Medicine Ethics Committee. All patients provided written informed consent for clinical investigation.

### Measurement and assessment of sHGF and carcinoembryonic antigen (CEA)

Serum was immediately separated from blood samples by centrifugation at 4°C and cryopreserved at −80°C until assays were performed. sHGF levels were measured at a medical laboratory testing company, SRL (Tokyo, Japan), using an enzyme-linked immunosorbent assay (ELISA) kit (Otsuka Pharmaceutical, Tokyo, Japan), which was designed and approved for use in diagnostic procedures. The mean ± S.D. level of sHGF in 200 adult Japanese healthy controls was 0.19 ± 0.05 ng/ml and the lower limit of detection had been determined to be 0.3 ng/ml, according to the manufacturer's data sheet [45]. sHGF values were categorized into 4 subgroups by time-points during the treatment; at pre-treatment, at response-evaluation (1–2 months after treatment initiation), at best tumor response, and at disease progression based on the Response Evaluation Criteria in Solid Tumors (RECIST) criteria ver. 1.1 [48]. As a control biomarker, the levels of serum CEA were also evaluated using ELISA. The values for sHGF were classified into 2 categories, Negative-sHGF and Positive-sHGF, with a threshold of 0.3 ng/ml since this is the lower limit of detection. The control biomarker, serum high-CEA, was defined in each subgroup as more than the median value of categorized subjects. First-line, second-line, first CC, and second CC treatments were defined in the conventional way (Supplementary Figure 1).

### sHGF in healthy control

The blood samples of healthy controls had been obtained in Kyoto University Hospital from August 2012 to December 2015 from volunteers without history of malignancy or hepatic disease. The serum was immediately separated and cryopreserved at −80°C without any freeze-thaw cycle until the assay was conducted. From the serum library, 30 healthy controls that were matched to the current study population by smoking status, gender, and age were collected. The sHGF value was evaluated using the same procedure as used for patients with NSCLC. Written informed consent had been obtained from these healthy controls at the time of blood sampling, and supplemental informed consent was obtained for using the serum in the current study. This healthy control study was also approved by the institutional ethics committee.

### Subgroup analysis

To assess the trend of sHGF values in the treatment course and the association between sHGF and clinical outcome, all patients were included for analysis. To assess the clinical predictive value of sHGF with CEA, patients with lung adenocarcinoma were exclusively included, because CEA, the control tumor marker, is a marker for adenocarcinoma, but not for other histology types in lung cancer.

### Statistical analysis

The efficacy was determined using the objective response rate (ORR), disease control rate (DCR), and PFS, in accordance with the RECIST criteria [48]. Differences in the distribution of variables were evaluated using Fisher's exact test or the chi-square test, as appropriate. sHGF values were not continuous variables and the difference was evaluated using the Wilcoxon matched-pairs signed rank test or the Mann-Whitney U test, as appropriate. PFS were estimated using the Kaplan–Meier Method, and survival curves were compared using the Gehan-Breslow-Wilcoxon or logrank test. Hazard ratios (HRs) and the corresponding 95% confidence intervals (CIs) for PFS were calculated using univariate analysis by the Cox proportional hazards model. In patients receiving cytotoxic chemotherapy, univariate Cox proportional hazards models were used to evaluate the associations between PFS and patient characteristics as follows; gender, age, smoking, ECOG performance status, stage, histology, EGFR-status, monotherapy, second-line treatment, and the sHGF value. Variables showing a univariate association with PFS (at *P* < 0.20) were included in stepwise multivariate Cox proportional hazards models. All tests were 2-sided, and a *P*-value of 0.05 was defined as significant. All statistical analyses were performed using JMP Pro version 12.0 (SAS Institute, Inc., Cary, NC, United States) and visualized by GraphPad Prism 6 (GraphPad software, La Jolla, CA, United States).

## SUPPLEMENTARY MATERIALS FIGURES AND TABLES



## Abbreviations

HGF: hepatocyte growth factor
NSCLC: non-small cell lung cancer
PFS: progression-free survival
ELISA: enzyme-linked immunosorbent assay
CEA: carcinoembryonic antigen
LOD: limit of detection
HR: hazard ratio
TKI: tyrosine kinase inhibitor
EGFR: epidermal growth factor receptor
SCLC: small-cell lung cancer
RECIST: Response Evaluation Criteria in Solid Tumors
CC: cytotoxic chemotherapy
MV: median value
