# Supplementary material for: Clinical impact of high serum hepatocyte growth factor in advanced non-small cell lung cancer
Source: Oncotarget. 2017 May 16;8(42):71805–16. doi: 10.18632/oncotarget.17895 (PMC5641091; doi:10.18632/oncotarget.17895)
Supplement: Supplementary file 1 [file oncotarget-08-71805-s001.pdf]

Clinical impact of high serum hepatocyte growth factor in advanced non-small cell lung cancer

Supplementary Materials

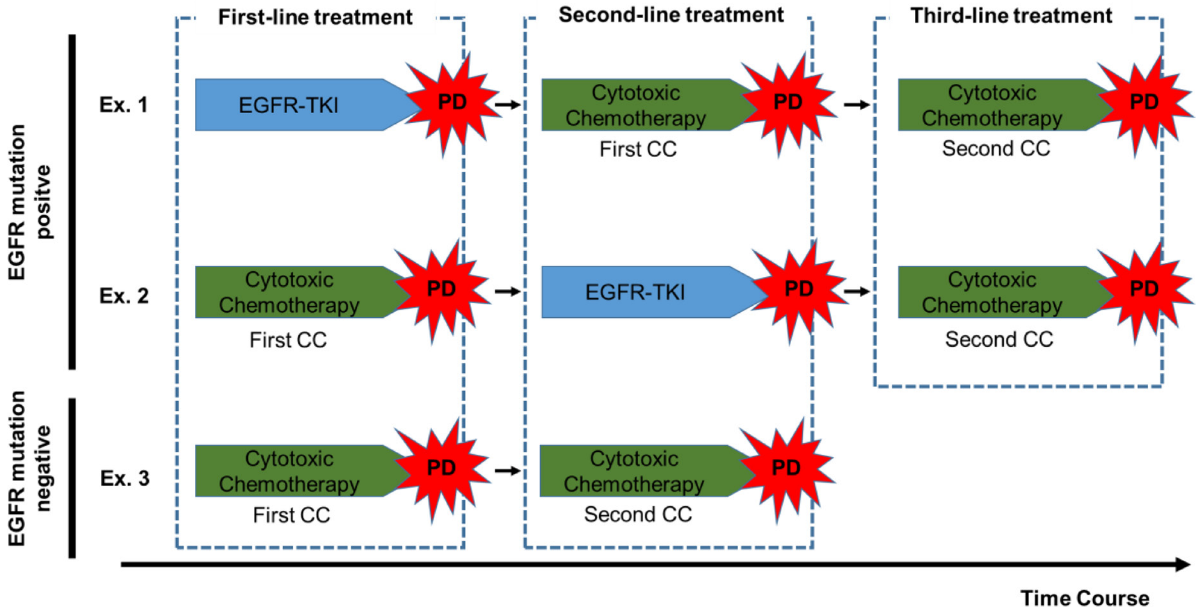

Supplementary Figure 1: Definition of treatment course. The treatment of first-line therapy was defined as the first treatment regimen in each patient, and second-line therapy was the subsequent treatment regimen after disease progression following first-line therapy. In lung adenocarcinoma, 3 major types of conventional treatment were clinically conducted. EGFR-TKIs are administrated as first-line or second-line treatments in patients with EGFR-mutated NSCLC (Ex. 1, 2), and subsequently cytotoxic chemotherapy is administrated as a third-line therapy. First cytotoxic chemotherapy was defined as the first cytotoxic chemotherapy regimen in each patient, which includes second-line cytotoxic chemotherapy after EGFR-TKIs, and second cytotoxic chemotherapy was the second administration of cytotoxic chemotherapy. EGFR: epidermal growth factor; TKI: tyrosine kinase inhibitor; CC: cytotoxic chemotherapy; PD: progressive disease.

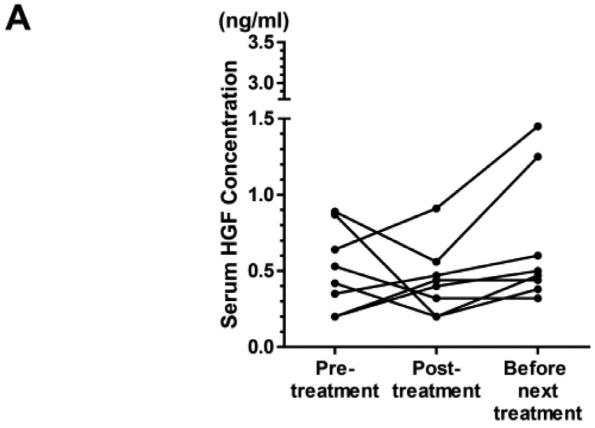

N	8		
Median	0.47	0.42	0.48
Increased from PreT	-	4	6
Decreased from PreT	-	4	2

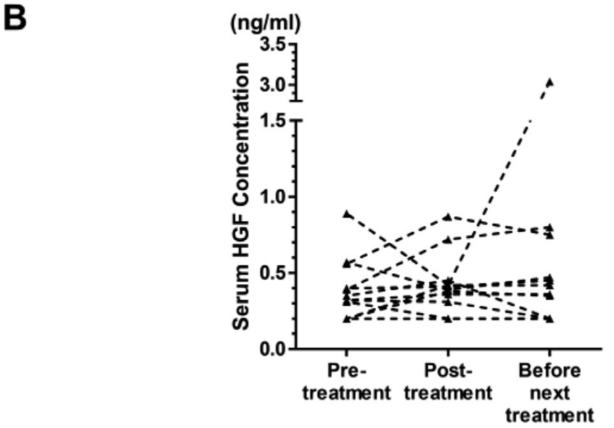

N	13		
Median	0.32	0.36	0.39
Increased from PreT	-	7	8
Decreased from PreT	-	5	4
No change		1	1

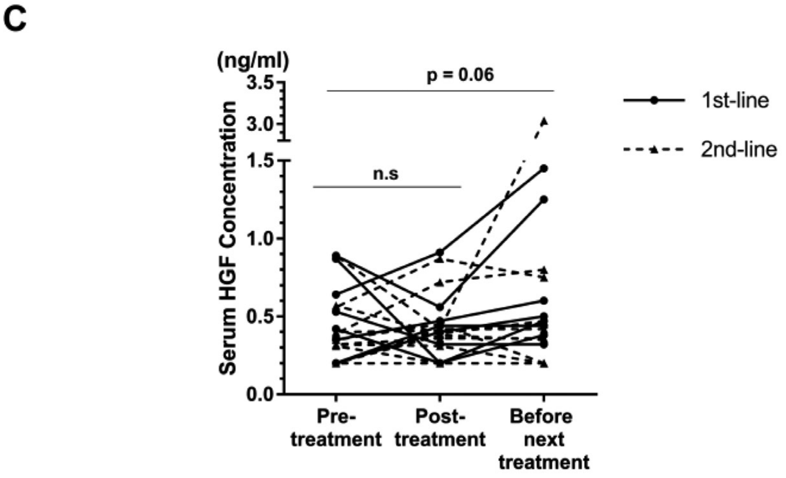

N	21		
Median	0.35	0.34	0.44
Increased from PreT	-	11	14
Decreased from PreT	-	9	6
No change		1	1

Supplementary Figure 2: sHGF levels in patients who diagnosed with PD at first response evaluation. (**A**) Serum hepatocyte growth factor (sHGF) trend in PD patients who received first-line (A) , or second-line (**B**), and in all patients (**C**). The Wilcoxon matched-pairs signed rank test was used for comparisons. DC: disease control; PD: progressive disease; LOD: limit of detection.

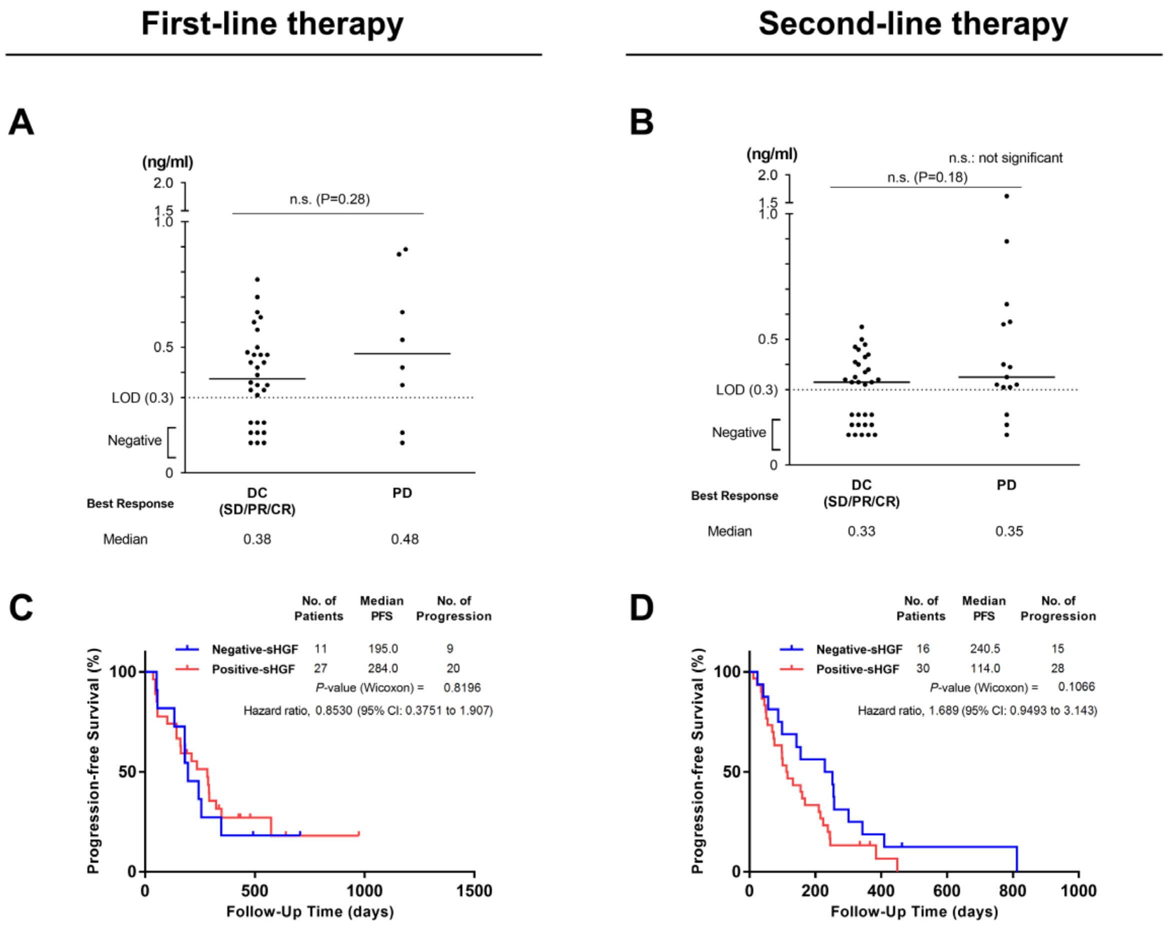

Supplementary Figure 3: sHGF levels at pre-treatment and progression-free survival both in first-line and second-line therapy. (**A**, **B**) Serum hepatocyte growth factor (sHGF) levels at pre-treatment according to disease controls receiving first-line (A) and second-line (B) therapy. Black circles indicate sHGF levels in each group, and the bars show median values. The P-value was calculated using the Mann Whitney *U* test. (**C**, **D**) A Kaplan-Meier curve for progression-free survival according to sHGF levels at pre-treatment in patients with NSCLC receiving first-line (C) and second-line (D) therapy. The P-value was calculated using the Gehan-Breslow-Wilcoxon test. DC: disease control; PD: progressive disease; LOD: limit of detection.

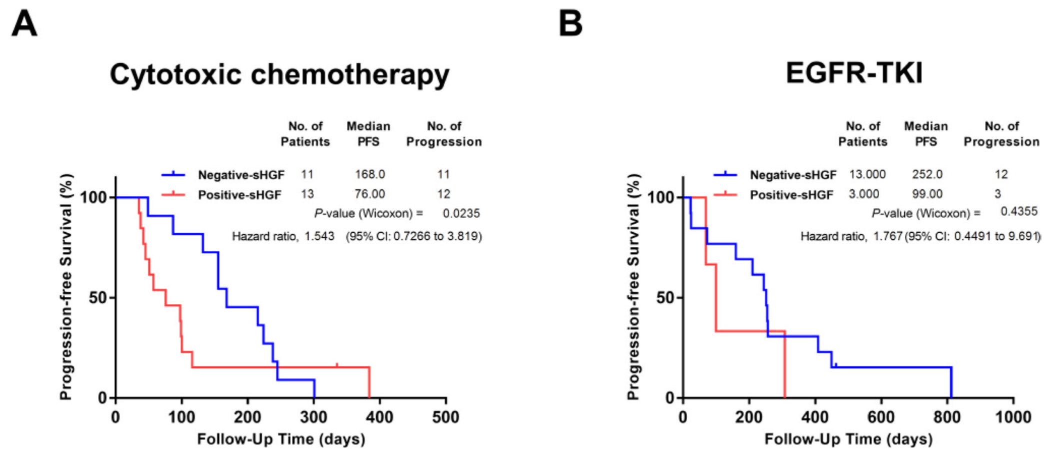

Supplementary Figure 4: Positive-sHGF at response-evaluation in second-line cytotoxic chemotherapy or EGFR-TKI tended to predict poor PFS. (**A**) Kaplan-Meier curve for progression-free survival according to sHGF levels at response-evaluation in patients with NSCLC receiving second-line cytotoxic chemotherapy (**B**) or in patients receiving second-line EGFR-TKI.

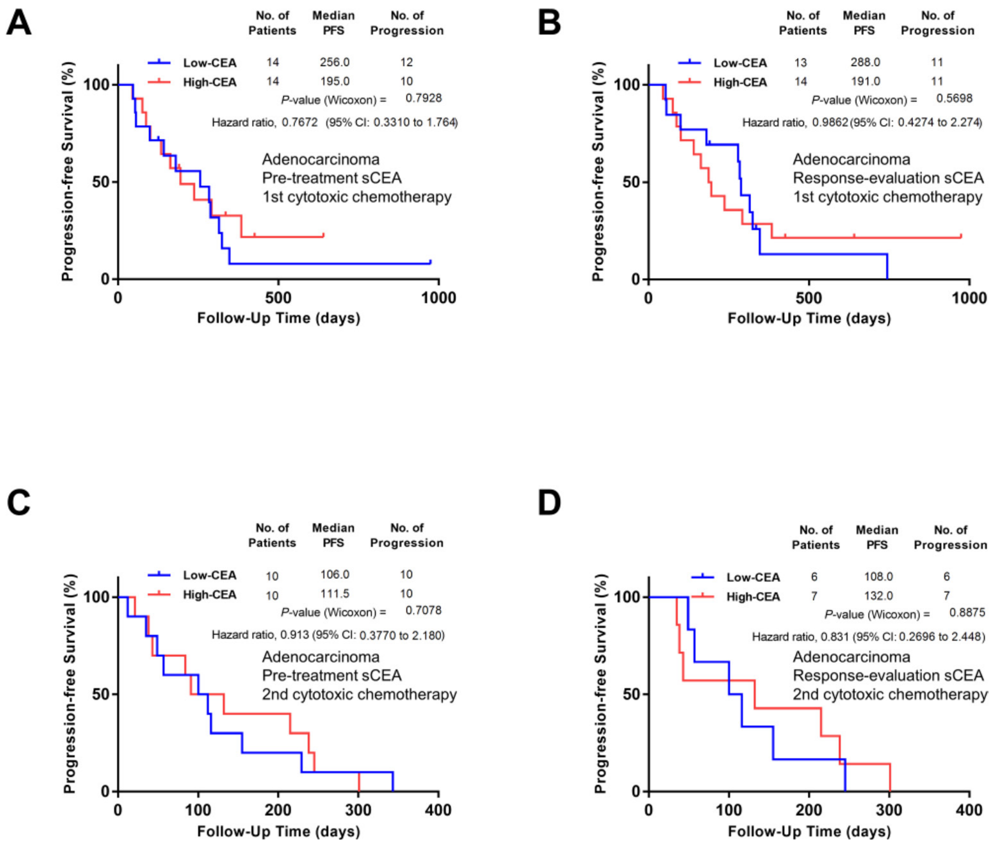

Supplementary Figure 5: Serum carcinoembryonic antigen (CEA) and PFS in patients with lung adenocarcinoma treated with cytotoxic chemotherapy. (**A**, **B**) A Kaplan-Meier curve for progression-free survival according to the CEA levels at pre-treatment (A) and at response-evaluation (B) in patients with lung adenocarcinoma receiving first cytotoxic chemotherapy. (**C**, **D**) A Kaplan-Meier curve for progression-free survival according to the CEA levels at pre-treatment (C) and at response-evaluation (D) in patients with lung adenocarcinoma receiving second cytotoxic chemotherapy. The *P*-value was calculated using the Gehan-Breslow-Wilcoxon test.

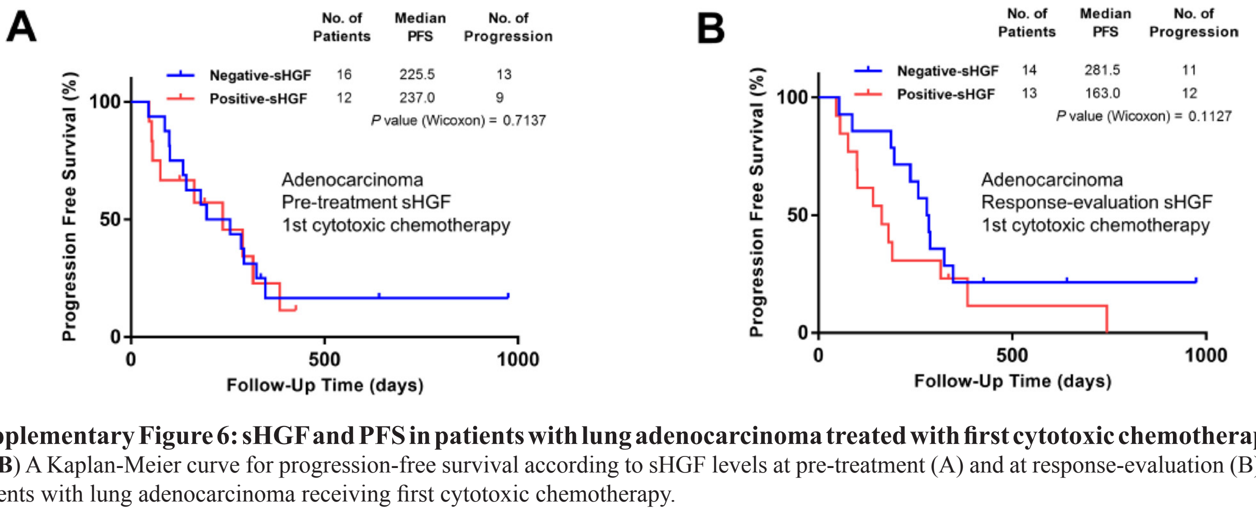

Supplementary Figure 6: sHGF and PFS in patients with lung adenocarcinoma treated with first cytotoxic chemotherapy. (**A**, **B**) A Kaplan-Meier curve for progression-free survival according to sHGF levels at pre-treatment (A) and at response-evaluation (B) in patients with lung adenocarcinoma receiving first cytotoxic chemotherapy.

Supplementary Table 1: A multivariate Cox proportional hazards model that includes five factors in patients that received cytotoxic chemotherapy

**Multivariate analysis**		
**Factor**	**HR (95% CI)**	***P*-value**
ECOG-PS (PS ≥ 2 / PS 0–1)	1.5 (0.33–4.91)	0.55
EGFR-status (WT or Sq / mutant)	7.97 (2.78–26.32)	**P* < 0.01
Monotherapy (Monotherapy / Platinum Doublet)	0.76 (0.32–1.86)	0.54
Second-line (2nd-line / 1st-line)	2.69 (1.16–6.03)	*0.02
sHGF at response-evaluation (Positive / Negative)	4.77 (2.32–10.1)	**P* < 0.01

HR: hazard-ratio; ECOG: Eastern Cooperative Oncology Group; PS: performance status; EGFR: epidermal growth factor receptor; WT: wild type; Sq: Squamous cell carcinoma; Ad: adenocarcinoma.

Supplementary Table 2: The characteristics of patients that received cytotoxic chemotherapy

***N* = 55**		**Monotherapy**	**Platinum Doublet**	***P*-value**
Treatment course	First-line	0	30	*< 0.01
	Second-line	15	10	

		Positive-HGF	Negative-HGF	*P*-value
N = 55		29	26	
Gender	Female	14 (48.3%)	8 (30.8%)	0.27
Age	<65	11 (37.9%)	14 (53.9%)	0.29
	≥65	18 (62.1%)	12 (46.2%)	
Smoking	Never Smoker	11 (37.9%)	9 (34.6%)	1.00
	Smoker	18 (62.1%)	17 (65.4%)	
Performance Status	PS ≥ 2	1 (3.5%)	2 (7.7%)	0.60
	PS 0–1	28 (96.6%)	24 (92.3%)	
Stage	III	4 (13.8%)	2 (7.7%)	0.67
	IV/recurrence	25 (86.2%)	24 (92.3%)	
Histology	Adenocarcinoma	22 (75.9%)	15 (57.7%)	0.24
	Non-adenocarcinoma	7 (24.1%)	11 (42.3%)	
EGFR-status	WT or Squamous cell carcinoma	24 (82.8%)	21 (80.8%)	1.00
	EGFR-mutant	5 (17.2%)	5 (19.2%)	
Monotherapy	Monotherapy	7 (24.1%)	8 (30.8%)	0.76
	Platinum Doublet	22 (75.9%)	18 (69.2%)	
Treatment course	First-line	17 (58.6%)	13 (50.0%)	0.59
	Second-line	12 (41.4%)	13 (50.0%)	

The backgrounds of patients from Table 3. PS: performance status; WT: wild type; EGFR: epidermal growth factor receptor.

Supplementary Table 3: The characteristics of patients that subsequently received anticancer treatment after progression during cytotoxic chemotherapy

	Pre-treatment		
	**Negative-HGF**	**Positive-HGF**	***P*-value**
N	15	15	
Female	9 (60.0%)	4 (26.7%)	0.14
Age, Mean ± S.D.	68.4 ± 9.9	60 ± 13.6	0.06
PS ≥ 2	2 (13.3%)	1 (6.7%)	1.00
PS 0–1	13 (86.7%)	14 (93.3%)	
Never Smoker	7 (46.7%)	5 (33.3%)	0.71
Smoker	8 (53.3%)	10 (66.7%)	
EGFR-positive	6 (40%)	6 (40%)	1.00
WT/Sq	9 (60%)	9 (60%)	
Cytotoxic chemotherapy	10 (66.7%)	11 (73.3%)	1.00
EGFR-TKI	5 (33.3%)	4 (26.7%)	
PR/SD	3/7 (66.7%)	2/8 (66.7%)	1.00
PD	5 (33.3%)	5 (33.3%)	

Data are presented as numbers. PS: performance status; EGFR: epidermal growth factor receptor; WT: wild type; Sq: squamous cell carcinoma; TKI: tyrosine kinase inhibitor; PR: partial response; SD: stable disease; PD: progressive disease.

Supplementary Table 4: The characteristics of patients with lung adenocarcinoma receiving first cytotoxic chemotherapy

	Pre-Treatment				Response-Evaluation			
	HGF		CEA		HGF		CEA	
	Negative-HGF	Positive-HGF	Low-CEA	High-CEA	Negative-HGF	Positive-HGF	Low-CEA	High-CEA
N	16	12	14	14	14	13	13	14
Female	6 (37.5%)	9 (75.0%)	7 (50.0%)	8 (57.1%)	9 (64.3%)	6 (46.2%)	7 (53.9%)	8 (57.1%)
Age, Mean ± SD	66.7 ± 10.5	63.8 ± 9.7	64.4 ± 10.9	66.4 ± 9.6	66.9 ± 6.4	58.6 ± 11.3	62.9 ± 10.4	62.9 ± 9.7
PS ≥ 2	0 (0%)	2 (16.7%)	1 (7.1%)	1 (7.1%)	1 (7.1%)	1 (7.7%)	1 (7.7%)	1 (7.1%)
PS 0–1	16 (100%)	10 (83.3%)	13 (92.9%)	13 (92.9%)	13 (92.9%)	12 (92.3%)	12 (92.3%)	13 (92.9%)
Never Smoker	5 (31.3%)	7 (58.3%)	7 (50.0%)	5 (35.7%)	6 (42.9%)	5 (38.5%)	5 (38.5%)	6 (42.9%)
Smoker	11 (68.8%)	5 (41.7%)	7 (50.0%)	9 (64.3%)	8 (57.1%)	8 (61.5%)	8 (61.5%)	8 (57.1%)
EGFR-positive	4 (25.0%)	5 (41.7%)	5 (35.7%)	4 (28.6%)	4 (28.6%)	6 (46.2%)	5 (38.5%)	5 (35.7%)
Unknown	2 (12.5%)	0 (0%)	1 (7.1%)	1 (7.1%)	2 (14.3%)	0 (0%)	1 (7.7%)	1 (7.1%)
Wild type	10 (62.5%)	7 (58.3%)	8 (57.1%)	9 (64.3%)	8 (57.1%)	7 (53.9%)	7 (53.9%)	8 (57.1%)
TKI history	3 (18.8%)	4 (33.3%)	4 (28.6%)	3 (21.4%)	1 (7.1%)	5 (38.5%)	3 (23.1%)	3 (21.4%)
PR	7 (43.8%)	3 (25.0%)	7 (50.0%)	3 (21.4%)	6 (42.9%)	5 (38.5%)	8 (61.5%)	3 (21.4%)
SD	8 (50.0%)	5 (41.7%)	4 (28.6%)	9 (64.3%)	7 (50.0%)	4 (30.8%)	3 (23.1%)	8 (57.1%)
PD	1 (6.3%)	4 (33.3%)	3 (21.4%)	2 (14.3%)	1 (7.1%)	4 (30.8%)	2 (15.4%)	3 (21.4%)

The characteristics of patients with lung adenocarcinoma and negative-HGF, positive-HGF, low-CEA, and high-CEA that received first cytotoxic chemotherapy. Data are presented as numbers. PS: performance status; EGFR: epidermal growth factor receptor; TKI: tyrosine kinase inhibitor; PR: partial response; SD: stable disease; PD: progressive disease.

Supplementary Table 5: The characteristics of patients with lung adenocarcinoma receiving second cytotoxic chemotherapy

	Pre-Treatment				Response-Evaluation			
	HGF		CEA		HGF		CEA	
	Negative-HGF	Positive-HGF	Low-CEA	High-CEA	Negative-HGF	Positive-HGF	Low-CEA	High-CEA
N	15	5	10	10	7	6	6	7
Female	7 (46.7%)	1 (20.0%)	5 (50.0%)	3 (30.0%)	3 (42.9%)	2 (33.33%)	3 (50.0%)	2 (28.6%)
Age, Mean ± SD	69.6 ± 7.8	57.0 ± 12.0	65.3 ± 9.2	67.6 ± 11.7	69.0 ± 8.8	70.2 ± 6.8	65.8 ± 6.0	72.7 ± 7.8
PS ≥ 2	2 (13.3%)	0 (0%)	1 (10.0%)	1 (10.0%)	0 (0%)	2 (33.3%)	1 (16.7%)	1 (14.3%)
PS 0–1	13 (86.7%)	5 (100%)	9 (90.0%)	9 (90.0%)	7 (100%)	4 (66.7%)	5 (83.3%)	6 (85.7%)
Never Smoker	6 (40.0%)	1 (20.0%)	3 (30.0%)	4 (40.0%)	3 (42.9%)	2 (33.3%)	2 (33.3%)	3 (42.9%)
Smoker	9 (60.0%)	4 (80.0%)	7 (70.0%)	6 (60.0%)	4 (57.1%)	4 (66.7%)	4 (66.7%)	4 (57.1%)
EGFR-positive	3 (20.0%)	1 (20.0%)	1 (10.0%)	3 (30.0%)	1 (14.3%)	1 (16.7%)	1 (16.7%)	1 (14.3%)
Unknown	1 (6.7%)	0 (0%)	1 (10.0%)	0 (0%)	0 (0%)	0 (0%)	0 (0%)	0 (0%)
Wild type	11 (73.3%)	4 (80.0%)	8 (80.0%)	7 (70.0%)	6 (85.7%)	5 (83.3%)	5 (83.3%)	6 (85.7%)
TKI history	3 (20.0%)	1 (20.0%)	1 (10.0%)	3 (30.0%)	1 (14.3%)	1 (16.7%)	1 (16.7%)	1 (14.3%)
PR	1 (6.7%)	0 (0%)	1 (10.0%)	0 (0%)	0 (0%)	0 (0%)	0 (0%)	0 (0%)
SD	10 (66.7%)	2 (40.0%)	5 (50.0%)	7 (70.0%)	6 (85.7%)	2 (33.3%)	4 (66.7%)	4 (57.1%)
PD	4 (26.7%)	3 (60.0%)	4 (40.0%)	3 (30.0%)	1 (14.3%)	4 (66.7%)	2 (33.3%)	3 (42.9%)

The characteristics of patients with lung adenocarcinoma and negative-HGF, positive-HGF, low-CEA, and high-CEA that received second cytotoxic chemotherapy. Data are presented as numbers. PS: performance status; EGFR: epidermal growth factor receptor; TKI: tyrosine kinase inhibitor; PR: partial response; SD: stable disease; PD: progressive disease.
